# Right anterolateral mini-thoracotomy without inferior vena cava cannulation for atrial septal defect repair in small children: A feasible technique

**DOI:** 10.1016/j.ijscr.2019.05.060

**Published:** 2019-06-10

**Authors:** Huy Q. Dang, Huu C. Nguyen, Huong T. Le, Thanh N. Le, Tuan Q. Nguyen

**Affiliations:** a Minimally Invasive Cardiac Surgery Unit, Cardiovascular Center, Hanoi Heart Hospital, Hanoi, Viet Nam; b Department of Cardiovascular and Thoracic Surgery, Cardiovascular Center, E Hospital, Hanoi, Viet Nam; c Institute of Preventive Medicine and Public Health, Hanoi Medical University (HMU), Hanoi, Viet Nam

**Keywords:** Right anterolateral mini-thoracotomy, ASD closure, Beating heart

## Abstract

**Objective:**

To evaluate the effectiveness and safety of right anterolateral mini-thoracotomy without inferior vena cava (IVC) cannulation for closing atrial septal defect (ASD) in small children.

**Methods:**

From February 2016 to August 2017, 10 patients (the mean age was 18.5 ± 10.1 months and the mean weight was 8.3 ± 2.1 kg) underwent ASD closure via right anterolateral mini-thoracotomy. The superior vena cava cannula was placed through the right internal jugular vein. A 3–4 cm incision was made on the right chest. The pleural and pericardial cavities were filled with CO_2_ and the heart was beating during the surgery. Blood returned from IVC was drained by a right heart sucker. All ASDs were closed using artificial patch, continuous suture. Mean follow-up was 18 months (range, 15–22 months).

**Results:**

No post-operative complications or deaths occurred. Mean operation time and mean cardiopulmonary bypass time were 140.5 ± 27.8 min and 50.3 ± 16.5 min, respectively. These patients were extubated within the first 6 h. The intensive care unit stay time and the post-operative hospital stay time were 19.6 ± 2.6 h and 7.1 ± 1.2 days, respectively. Follow-up transthoracic echocardiography showed no residual shunts or lung atelectasis.

**Conclusions:**

The right anterolateral mini-thoracotomy without IVC cannulation is feasible for repairing ASD in small children. This technique is effective and safe and can be used as a therapeutic option for ASD.

## Highlights

• Minimally invasive cardiac surgery is limited in small children.
• Femoral cannulation has high risks in children with body weight <15 kg.
• 7–12 cm skin incision called “mini-thoracotomy” was used with less cosmetic results.
• Reducing the length of incision without femoral cannulation needs new approach.
• This technique is safe and feasible for atrial septal defect repair.

## 1 Introduction

Atrial septal defect (ASD) is one of the most common congenital heart disease [1], and sometimes needs to be closed during childhood. There are some reports on the application of totally endoscopic surgery (TES) for ASD repair in children with the weight threshold being 13.5 kg [2,3]. However, femoral arterial (FA) cannulation may predispose these small patients to certain risks: (1) acute lower limb ischaemia, and (2) post-operative stenosis of the iliac or femoral arteries [4,5]. We reported on using transthoracic aortic cannulation for totally endoscopic ASD repair in a 10.5-kg-boy [4], but it seems impossible for smaller children. Some authors favored right thoracotomy (about 12 cm in length) with all cannulae set up directly through the incision [[6], [7], [8]]. Despite of the safety and the effectiveness, this approach with a long incision was not much cosmetic and resulted in risks of breast or chest wall mal-development [8]. We report our first experience in repairing ASD in small children via right anterolateral mini-thoracotomy with a new method of setting up cannulae.

## 2 Patients and methods

### 2.1 Patient selection

We selected patients with the following inclusion criteria: (1) isolated secundum ASD, ASD associated with partial anomalous pulmonary venous connection (p-APVC), (2) body weight ≤13 kg, and (3) no history of operation on the right lung. From February 2016 to August 2017, 10 patients who met all of the criteria underwent ASD repair through right anterolateral mini-thoracotomy in our institute. The mean age was 18.5 ± 10.1 months (range, 8–42 months) and the mean weight was 8.3 ± 2.1 kg (range, 6.4–12 kg). Pre-operative demographic indices (age, weight, skin area, defect size), characteristics of pathophysiology and indications for surgery are presented in Table 1. Surgical technique was approved by Scientific Board of the Hospital and by patients’ family. The research work has been reported in line with the PROCESS criteria [9].

**Table 1:** Demographics and preoperative indices (n = 10).

Age (months)		18.5 ± 10.1 (8–42)
Sex (Male/Female)		3/7
Weight (kg)		8.3 ± 2.1 (6.4–12)
Skin area (m^2^)		0.4 ± 0.05 (0.35–0.5)
Types of disease		
	Secundum ASD	9
	ASD combined with p-APVC	1
Size of the defect (mm)		18.8 ± 5.6 (14–29)
Right ventricle (mm)		20.4 ± 4.1 (15–27)
Indications for surgery		
	Failed trans-catheter closure	0
	Multiple defects	3
	No edge	3
	Short, thin edge	3
	Anomalous pulmonary venous return	1

**ASD**: atrial septal defect, **p-APVC**: partial anomalous pulmonary vein connection.

### 2.2 Anesthesia

**Fig. 1:**
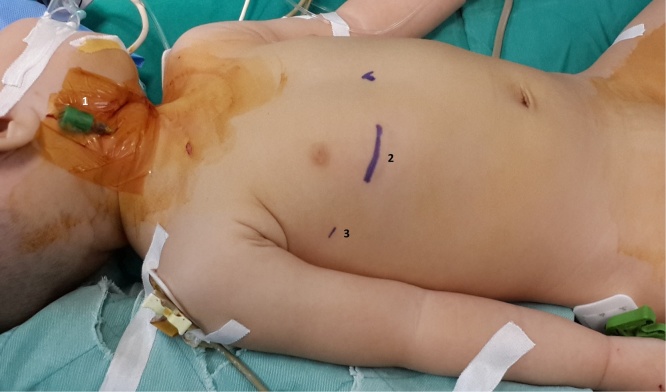
Anesthesia and marking of the incision. (1) A needle was placed into the right internal jugular vein (prepared for superior vena cava cannulation), (2) 3–4 cm incision for right anterolateral mini-thoracotomy, (3) incision for 5 mm trocar.

The anesthetist placed the central venous catheter through left internal jugular vein (IJV) and inserted a needle – which would be subsequently used for guide-wire introduction for superior vena cava (SVC) cannulation – in the right IJV (Fig. 1); all procedures were performed under sterile condition.

### 2.3 Surgical technique

The patient was positioned in the supine position with the right side of the body elevated to 20–30^°^, two hands were placed along the body, and patient’s head were tilted to the left to expose the already placed intravenous needle (Fig. 1). The SVC cannula was placed through the right IJV with Seldinger technique.

**Fig. 2:**
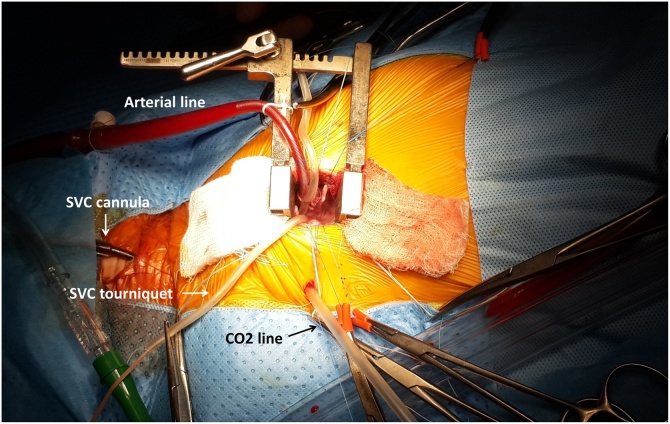
Intraoperative view of right anterolateral mini-thoracotomy for ASD repair. The superior vena cava (SVC) cannula was placed through the right internal jugular vein with Seldinger technique. The chest was entered through the fourth intercostal space (ICS). One 5 mm trocar was placed at the fourth ICS in the mid-axillary line (for CO_2_ line). The pleural and pericardial cavities were filled with CO_2_ which was pumped at the rate of 0.5 l/min. Aortic cannulation was performed through the incision and the cannula was fixed on the retractor. A tourniquet was placed around the SVC.

A skin incision of about 3–4 cm was made well below the nipple. The chest was entered through the fourth intercostal space (ICS). One 5 mm trocar was placed at the fourth ICS in the mid-axillary line (for tissue forceps or CO_2_ line) (Fig. 2). The right lobe of the thymus always covered the majority of the pericardium surrounding the aorta and the SVC. Therefore, we dissected this lobe from the pericardium (while preserving the tissue and supplying vessels) and hung it on to the anterior chest wall with a suture. The pericardium was opened parallel to and at 1.5 cm away from the anterior chest wall. The pleural and pericardial cavities were filled with CO_2_ which was pumped at the rate of 0.5 l/min.

To expose the ascending aorta, the top of the right atrial appendage was sutured and pulled down. A 4–0, 17 mm braided suture (ETHIBOND EXCEL^®^ Polyester Suture, ETHICON, JOHNSON & JOHNSON, Shanghai, China) was used to make a purse-string suture on the anterior wall of the ascending aorta, right beneath the semicircular fat plica. An arterial cannula (Medtronic, Inc., Minneapolis, Minn, USA) was placed superiorly through the incision into the ascending aorta (Fig. 2). We placed a piece of a 10Fr rubber catheter (Red Rubber Latex All-Purpose Intermittent Catheters, Medline, USA) about 1.3–1.5 cm away from the tip of the arterial cannula to work as a brake. The arterial cannula was fixed and the cardiopulmonary bypass (CPB) was started. The patient was placed in the Trendelenburg position and the arterial line pressure was kept ≥50 mmHg during the operation.

Right after snaring the SVC, the right atrium (RA) was opened. Heart continuously beat during surgery. Blood flowed to operation field through the coronary sinus, inferior vena cava (IVC) ostium, and the direct ostia on RA wall. A right heart sucker was used to remove blood from the surgical field and was used as a retractor to expose the defect. To hit these marks, the tip of this sucker should be put at the IVC ostium. The speed of the suction pump was controlled to minimize co-aspiration of air. All defects were closed with an artificial patch (Neuro-patch; Aesculap AG, Tuttlingen, Germany), continuous suture. Anomalous pulmonary veins were drained to left atrium through ASD. Right before the completion of ASD repair, the lung was briefly inflated to remove air from the left atrium. The RA was closed in a two-layer fashion using continuous stitches (Table 2).

**Table 2:** Parameters during and after operation (n = 10).

Operation time (min)	140.5 ± 27.8 (90–180)
CPB time (min)	50.3 ± 16.5 (29–72)
Setting up CPB time (min)	33.1 ± 3.3 (30–40)
Ventilation time (h)	5.8 ± 1.3 (4–8)
ICU stay (h)	19.6 ± 2.6 (18–24)
Drainage volume in the first 24 h (ml)	13.3 ± 8.7 (10–30)
Postoperative hospital stay (day)	7.1 ± 1.2 (5–8)
Residual ASD	0
Hemolysis, Atelectasis, slow surgical wound healing,…	0
Extend the incision or convert to sternotomy	0

**ASD**: atrial septal defect; **CPB**: cardiopulmonary bypass; **ICU**: intensive care unit.

Heart was filled after removing SVC snare and placing patient in supine position. CPB was stopped and all the cannulae were taken off. One pericardial and one pleural drain were placed through one trocar. The pericardium was closed and the remaining procedures were similar to normal video-assisted surgery.

### 2.4 Post-operative follow-up

Follow-up appointments were scheduled at 1 month, 3 months, 6 months and 1 year after surgery. Patients were examined with transthoracic echocardiography.

### 2.5 Statistical analysis

Data are expressed as mean ± SD for quantitative variables and number and percentage for qualitative variables. Data are managed and analyzed with SPSS 15.0 software.

## 3 Results

All patients had successful ASD repair. All procedures were performed by the same surgeon. There was no mortality or serious complications. No incision extension or conversion to median sternotomy was needed, and no patient underwent reoperation for bleeding. The FA cannulae (Medtronic, Inc., Minneapolis, Minn, USA) were used for both SVC and aortic cannulation (10–14Fr for SVC and 12–14Fr for aorta). Artificial patch was used for all of 10 patients and 1 patient underwent ASD repair combined with draining blood from anomalous pulmonary vein to the left atrium.

The mean operation time and CPB time were 140.5 ± 27.8 min (range, 90–180 min) and 50.3 ± 16.5 min (range, 29–72 min), respectively. All patients did not need to use vasoactive drugs after stopping CPB and were extubated within the first 6 h. Patients stayed in the intensive care unit (ICU) for about 1 day and discharged after 1 week of the operation.

**Fig. 3:**
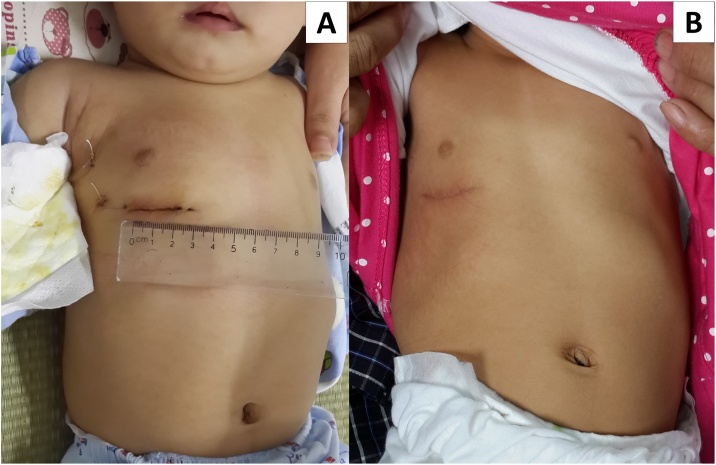
The patients after operation. (A) a 7.5-kg-boy with a 3 cm length incision before discharge; (B) a 6.8-kg-girl at follow-up of 6 months.

No peri-operative neurological events were recorded. No residual ASD was detected on post-operative transthoracic echocardiography performed before discharge. All patients were discharged in good condition and cosmetic results were excellent (Fig. 3). There were no major postoperative complications during the follow-up period.

## 4 Discussions

There are 3 methods of minimally invasive cardiac surgery for ASD repair in small children: (1) TES or mini-thoracotomy using femoral arterial (FA) cannulation, (2) TES using trans-thoracic aortic cannulation, and (3) thoracotomy using aortic cannulation through incision.

Ma et al. [10] and Wang et al. [3] reported on direct FA cannulation, while we [2,11] reported on indirect FA cannulation via No6-Knitted Dacron graft (Vascutek Terumo, Bangkok, Thailand) for children from 13.5 kg. However, direct FA cannulation was associated with a rate of peripheral vascular complication of 0.68–2.48% with 63.6% presenting at long-term follow-up [5,12,13]. Muhs et al. [5] noted that small FA was the risk factor (p = 0.07) of iliofemoral arterial occlusion or stenosis. In fact that nobody had much experience in small children; we believe that this complication might be higher in this group of patients. Theoretically indirect FA cannulation seems to be safer due to not intervene in external iliac artery and due to leave a piece of Dacron graft to prevent FA stenosis, but this technique need longer follow-up duration to demonstrate its safety.

It is common knowledge that aortic cannulation should be applied for patients who are thought to be at increased risk for complications from femoral cannulation [5]. We published a case report of totally endoscopic ASD repair using trans-thoracic aortic cannulation in a 10.5-kg-boy [4]. Nevertheless, it’s difficult to use this technique safely in smaller children. While Dabritz et al. [6] and Panos et al. [7] used anterolateral thoracotomy, Sunil et al. [8] used posterior thoracotomy to close ASD for patients from 10 kg. In these reports, all of the cannulae for aorta, SVC and IVC were set up through a large incision (around 12 cm in length). In spite of the advantages of safety and short operation time, authors worried about long-term breast and chest wall maldevelopment in prepubertal patients of this approach [14].

For the reasons above, median sternotomy is the standard surgical approach for ASD closure in small children, especially in children <10 kg.

Our approach had better cosmetic result and lower risk of breast maldevelopment in comparison with thoracotomy approach which mentioned above. However, ASD closure through small incision (3–4 cm) seem to be impossible if (1) all the cannulae are set up through this incision and (2) surgery is performed with myocardial protection. In order to optimize the working space, we made some changes: (1) SVC cannula was placed through the right IJV with Seldinger technique, (2) a right heart sucker was used to attract blood returning from the IVC and was used as a retractor to expose the defect, and (3) surgery on the beating heart. As the result, we had the most of the operating field for manipulation. The CPB time in our report was equivalent to that in reports of Dabritz et al. [6] and Sunil et al. [8] (thoracotomy approach) and half as long as that of totally endoscopic ASD repair [15].

The major concerns of our method are the prevention of air embolism and risk of hemolysis. We used following principles to prevent air embolism: (1) keep left atrium to be always filled with blood by only putting the tip of right heart sucker at IVC ostium, (2) maintain the CPB perfusion pressure greater than 50 mmHg, (3) inflate the lung right before completing the ASD closure to remove air from the left atrium, and (4) fill the pericardial and pleural space with CO_2_. The CPB perfusion pressure which was greater than 50 mmHg was recommended in children to keep aortic valve from opening [16]. CO_2_ which has strong ability to dissolve in water has been shown to play an important role in preventing air embolism and can replace aortic root needle [17,18]. With these principles, we have successfully performed ASD repair on beating heart for more than 150 patients (included small children and adults). Among them, no peri-operative neurological events were recorded.

Hemolysis, depending on the severity, can cause systemic changes and make the condition of patients worse [19]. In our study, risk of hemolysis should be thought about because of using roller pump to drain blood returning from IVC into the reservoir. The mechanisms of hemolysis during CPB include negative pressure, blood–air interface, shear stress, time of exposure [20,21]. Leverett et al. [20] noted that shear stress was the major factor for red blood cell damage. It has been suggested to set the pump as non-occlusive as possible to decrease tubing spallation, increase tubing life and minimizing hemolysis [19]. In this study, we didn’t experience hemolysis due to the following factors: (1) ensure the roller pump worked in a non-occlusive condition, (2) because of low body weight, the blood flow from the lower part of the body which was drained through the roller pump was not much, (3) control the speed of the suction pump to minimize co-aspiration of air, and (4) short duration of using high suction rate (time for closing ASD, from 20 to 30 min). Vercaemst et al. [19] emphasized that blood flow drained through roller pump at rates from 100 to 700 mL/min and minimum of air intake are keys resulting in significantly less hemolysis.

Because of small incision, it’s difficult to repair tricuspid valve if necessary. Fortunately, ASDs in small children is not always combined with tricuspid valve regurgitation. Small sample size with lack of long-term follow-up are also limitations of this case series. In our opinions, for patients greater than 15 kg, the IVC cannula should be placed via femoral vein and totally endoscopic ASD closure should be done if possible.

## 5 Conclusions

The right anterolateral mini-thoracotomy without IVC cannulation is feasible for repairing ASD in small children. This technique is effective and safe and can be used as a therapeutic option for ASD.

## Conflicts of interest

No conflict of interest declared.

## Funding source

No funding was received for the study.

## Ethical approval

Ethical approval is not needed in Vietnam.

## Consent

Written informed consents were obtained from the parents of the patient for publication of these case report and accompanying images. We have consent of each patient. The copies of the written consents are available for review by the Editor-in-Chief of this journal on request.

## Author’s contribution

All authors have taken part in conception of the study, drafting and revising the whole manuscript critically. All authors have given their final approval of the manuscript upon submission.

## Registration of research studies

Researchregistry.

UIN: researchregistry4747.

## Guarantor

Huy Q. Dang.

## Provenance and peer review

Not commissioned, externally peer-reviewed.

## CRediT authorship contribution statement

**Huy Q. Dang:** Conceptualization, Methodology, Formal analysis, Writing - original draft, Writing - review & editing. **Huu C. Nguyen:** Methodology, Investigation. **Huong T. Le:** Software, Validation, Formal analysis. **Thanh N. Le:** Resources, Supervision. **Tuan Q. Nguyen:** Visualization, Supervision.

## References

[1] van der Linde D., Konings E.E., Slager M.A., Witsenburg M., Helbing W.A., Takkenberg J.J. (2011). Birth prevalence of congenital heart disease worldwide: a systematic review and meta-analysis. J. Am. Coll. Cardiol..

[2] Dang Q.-H., Le N.-T., Nguyen C.-H., Tran D.-D., Nguyen D.-H., Nguyen T.-H. (2017). Totally endoscopic cardiac surgery for atrial septal defect repair on beating heart without robotic assistance in 25 patients. Innov. Technol. Tech. Cardiothorac. Vasc. Surg..

[3] Wang F., Li M., Xu X., Yu S., Cheng Z., Deng C. (2011). Totally thoracoscopic surgical closure of atrial septal defect in small children. Ann. Thorac. Surg..

[4] Dang H.Q., Le H.T., Ngo L.T.H. (2018). Totally endoscopic atrial septal defect repair using transthoracic aortic cannulation in a 10.5-kg-boy. Int. J. Surg. Case Rep..

[5] Muhs B.E., Galloway A.C., Lombino M., Silberstein M., Grossi E.A., Colvin S.B. (2005). Arterial injuries from femoral artery cannulation with port access cardiac surgery. Vasc. Endovasc. Surg..

[6] Dabritz S., Sachweh J., Walter M., Messmer B.J. (1999). Closure of atrial septal defects via limited right anterolateral thoracotomy as a minimal invasive approach in female patients. Eur. J. Cardio-Thorac. Surg..

[7] Panos A., Aubert S., Champsaur G., Ninet J. (2003). Repair of atrial septal defect through a limited right anterolateral thoracotomy in 242 patients: a cosmetic approach?. Heart Surg. Forum.

[8] Sunil G.S., Koshy S., Dhinakar S., Shivaprakasha K., Rao S.G. (2002). Limited right posterior thoracotomy approach to atrial septal defect. Asian Cardiovasc. Thorac. Ann..

[9] Agha R.A., Borrelli M.R., Farwana R., Koshy K., Fowler A.J., Orgill D.P. (2018). The PROCESS 2018 statement: updating consensus preferred reporting of case series in surgery (PROCESS) guidelines. Int. J. Surg. (Lond., Engl.).

[10] Ma Z.S., Dong M.F., Yin Q.Y., Feng Z.Y., Wang L.X. (2011). Totally thoracoscopic repair of atrial septal defect without robotic assistance: a single-center experience. J. Thorac. Cardiovasc. Surg..

[11] Dang H.Q., Le T.N., Ngo L.T.H. (2018). Totally endoscopic surgical repair of partial atrioventricular septal defect in children: two cases. Innovations (Philadelphia, Pa).

[12] Jeanmart H., Casselman F.P., De Grieck Y., Bakir I., Coddens J., Foubert L. (2007). Avoiding vascular complications during minimally invasive, totally endoscopic intracardiac surgery. J. Thorac. Cardiovasc. Surg..

[13] Sagbas E., Caynak B., Duran C., Sen O., Kabakci B., Sanisoglu I. (2007). Mid-term results of peripheric cannulation after port-access surgery. Interact. Cardiovasc. Thorac. Surg..

[14] Cherup L.L., Siewers R.D., Futrell J.W. (1986). Breast and pectoral muscle maldevelopment after anterolateral and posterolateral thoracotomies in children. Ann. Thorac. Surg..

[15] Yao D.K., Chen H., Ma L.L., Ma Z.S., Wang L.X. (2013). Totally endoscopic atrial septal repair with or without robotic assistance: a systematic review and meta-analysis of case series. Heart Lung Circ..

[16] Mo A., Lin H., Wen Z., Lu W., Long X., Zhou Y. (2008). Efficacy and safety of on-pump beating heart surgery. Ann. Thorac. Surg..

[17] Chaudhuri K., Storey E., Lee G.A., Bailey M., Chan J., Rosenfeldt F.L. (2012). Carbon dioxide insufflation in open-chamber cardiac surgery: a double-blind, randomized clinical trial of neurocognitive effects. J. Thorac. Cardiovasc. Surg..

[18] Landenhed M., Al-Rashidi F., Blomquist S., Hoglund P., Pierre L., Koul B. (2014). Systemic effects of carbon dioxide insufflation technique for de-airing in left-sided cardiac surgery. J. Thorac. Cardiovasc. Surg..

[19] Vercaemst L. (2008). Hemolysis in cardiac surgery patients undergoing cardiopulmonary bypass: a review in search of a treatment algorithm. J. Extra. Technol..

[20] Leverett L.B., Hellums J.D., Alfrey C.P., Lynch E.C. (1972). Red blood cell damage by shear stress. Biophys. J..

[21] Wright G. (2001). Haemolysis during cardiopulmonary bypass: update. Perfusion.

